# Challenges in Teledermoscopy Diagnostic Outcome Studies: Scoping Review of Heterogeneous Study Characteristics

**DOI:** 10.2196/60346

**Published:** 2024-10-18

**Authors:** Femke van Sinderen, Anne P Langermans, Andre W Kushniruk, Elizabeth M Borycki, Monique M Jaspers, Linda W Peute

**Affiliations:** 1 Department of Medical Informatics Amsterdam UMC location University of Amsterdam Amsterdam Netherlands; 2 Digital Health Amsterdam Public Health Amsterdam Netherlands; 3 Ksyos Health Management Research Amsterdam Netherlands; 4 School of Health Information Science University of Victoria Victoria, BC Canada

**Keywords:** teledermatology, teledermoscopy, dermatologist, dermatology, telemedicine, e-health, telehealth, scoping review, heterogeneity, variability, diagnostic, content analysis, mobile phone

## Abstract

**Background:**

Teledermoscopy has demonstrated benefits such as decreased costs and enhanced access to dermatology care for skin cancer detection. However, the heterogeneity among teledermoscopy studies hinders the systematic reviews’ synopsis of diagnostic outcomes, impeding trust and adoption in general practice and limiting overall health care benefits.

**Objective:**

This study aims to improve understanding and standardization of teledermoscopy diagnostic studies, by identifying and categorizing study characteristics contributing to heterogeneity. Subsequently, the variability and consistency of these characteristics were assessed.

**Methods:**

A review of systematic reviews regarding the diagnostic outcomes of teledermoscopy was performed to discern reported study characteristics contributing to heterogeneity. These characteristics were thematically grouped into 3 domains (population, index test, and reference standard), forming a data extraction framework. A scoping review on teledermoscopy diagnostic outcomes studies was performed, guided by the PRISMA-ScR (Preferred Reporting Items for Systematic Reviews and Meta-Analyses extension for Scoping Reviews) checklist. Data pertaining to study characteristics from included studies were extracted and analyzed through descriptive content analysis. Systematic reviews’ reference lists validated the scoping review query.

**Results:**

The literature search yielded 4 systematic reviews, revealing 15 heterogeneous studies across the population, index test, and reference standard domains. The scoping review identified 49 studies, with 27 overlapping with the systematic reviews. Population characteristics varied, with one-third (16/49, 33%) of studies reporting fewer than 100 samples; most studies (41/49, 84%) reported on the type of lesion, and most (20/49, 41%) teledermoscopy consultations took place in secondary care. One-fifth (11/49, 22%) did not describe inclusion or exclusion criteria, or the criteria varied highly. Index test characteristics showed differences in clinical expertise, profession, and training in dermatoscopic photography, and 59% (29/49) did not report on 1 or more index test characteristics. Image quality and clinical information reporting likewise varied. Reference standard characteristics involved teledermatologists’ assessment, but 16 studies did not report teledermatologists’ experience levels. Most studies (26/49, 53%) used histopathology as a gold standard.

**Conclusions:**

The heterogeneity in the population, index tests, and reference standard domains across teledermoscopy diagnostic outcome studies underscores the need for standardized reporting. This hinders the synopsis of teledermoscopy diagnostic outcomes in systematic reviews and limits the integration of research results into practice. Adopting a (tailored) STARD (Standards for Reporting Diagnostic Accuracy Studies) checklist for teledermoscopy diagnostic outcome studies is recommended to enhance the consistency and comparability of outcomes. We suggest performing a Delphi study to gather consensus on the tailored STARD guideline.

## Introduction

Teledermoscopy is a telemedicine application used to diagnose potential malignant lesions by remote dermatologists. This technology supports physicians in the detection of skin cancer [1,2]. Over the past few years, numerous studies and (systematic) reviews have been conducted to study teledermoscopy, highlighting benefits, such as decreased overall costs and enhanced access to dermatology care, for remote patients [3-5]. Besides these benefits, ensuring the diagnostic accuracy of teledermoscopy is of utmost importance for safe and reliable care. Early detection of skin cancer is crucial, as late detection or unidentified cancer increases the risk for metastasis and worsens survival outcomes [6]. With the increasing incidence of skin cancers, it is therefore important that (systematic review) studies accurately reflect diagnostic outcomes related to teledermoscopy [7].

Various studies and systematic reviews have investigated the diagnostic outcomes of teledermoscopy, which are defined as the accuracy of a diagnostic test compared with a gold standard (eg, histopathology). Although numerous studies reported positive diagnostic outcomes of teledermoscopy, systematic reviews state that a synopsis is still absent. For example, Chuchu et al [8] reported that sensitivities ranged widely from 59% (95% CI 42%-74%) to 100% (95% CI 48%-100%) for the detection of invasive melanoma and melanocytic variants. This variability is likely attributed to insufficient methodological quality and diverse study designs, making it challenging to derive a single reliable estimate of the diagnostic outcomes. A total of 5 (Cochrane) systematic reviews ascribe this heterogeneity to a variety of other factors, including variations in study characteristics, such as the complexity in the detection of certain skin lesion types [8-12]. In addition to these teledermoscopy studies, a recent systematic review focusing solely on teledermatology reported that the included studies were too heterogeneous for significant conclusions about the diagnostic agreement. This was proven by subgroup analysis to control for confounding factors (eg, training for image acquisition). These results reveal the heterogeneity among diagnostic outcome studies, which hinders conducting a meta-analysis [8-12]. Moreover, a scoping review on consensus guidelines for teledermatology highlights the need for updating guidelines and thereby incorporating lessons from the COVID-19 pandemic [13]. While many guidelines emerged during the pandemic addressing specific issues, such as staff shortages and quarantine measures, there is a lack of new guidance on emerging technologies and postpandemic practices. This underscores the need to understand to what extent the characteristics of teledermoscopy studies differ before findings can be translated into practice guidelines. Therefore, this review aims to identify and categorize study characteristics that have been reported for contributing to heterogeneity in teledermoscopy diagnostic outcome studies. Subsequently, the variability and consistency of these study characteristics in the reporting of teledermoscopy diagnostic outcome studies were assessed.

## Methods

### Literature Search

A comprehensive search query was developed in collaboration with a medical librarian, including keywords, such as telemedicine, teledermatology, teleconsultation, teledermoscopy, and relevant medical conditions (Multimedia Appendix 1). The literature search was conducted in PubMed (January 1, 2023) [14]. Following the initial search, duplicate references were identified and excluded. We adhered to the PRISMA-ScR (Preferred Reporting Items for Systematic Reviews and Meta-Analyses extension for Scoping Reviews) guidelines for reporting (Multimedia Appendix 2) [15]. Initially, a review of systematic reviews was performed before we continued with the reference selection of our scoping review.

### Review of Systematic Reviews

The cohort of references was searched for systematic reviews by screening the titles and abstracts. Systematic reviews were subject to further analysis when the title or abstract explicitly mentioned the term “systematic review.” Upon full evaluation, systematic reviews were included if they aimed to assess the diagnostic outcomes of teledermoscopy performed by health care providers. An in-depth assessment of the included systematic reviews was then performed to retrieve study characteristics contributing to heterogeneity among the teledermoscopy diagnostic outcome studies. These identified study characteristics were then extracted and thematically organized into associated domains, including a description of the patient population (eg, sample size and selected skin lesion), features of the index test (eg, profession of the photographer), and aspects related to the reference standard (eg, profession of the assessor of the teledermoscopy consultation). This resulted in a structured framework of these study characteristics for the subsequent analysis of the included references of the scoping review.

### Scoping Review

Two authors (FvS and APL) independently evaluated the title, abstract, and full papers of the cohort of references resulting from the literature search. In case of inconsistencies between the evaluations, collaborative discussions were initiated between the 2 authors to reach a consensus. A third, independent author (LWP) was involved in the discussion if needed.

Initially, the references were selected by title, requiring relevance to teledermoscopy, teledermatology, suspicious skin lesions, or an imaging technique to be eligible for the subsequent abstract and full paper evaluation. Titles lacking this relevance were directly excluded, especially those that were clearly not an original study (eg, “a systematic review”).

Subsequent exclusion criteria for the abstract and full papers were drawn up by 2 authors (FvS and APL) in a discussion meeting, with input from a third author (LWP). References were excluded after reading the abstract and full papers as follows:

1. A dermatoscopic picture was not made (ie, the picture was not made with a digital dermatoscope or mobile phone with a dermatoscope attachment).
2. The domain was not teledermoscopy for the detection and management of skin lesions (eg, psoriasis, teledermatopathology, or the goal of the study is education or cost-benefit analysis).
3. There was no health care provider involved in the teledermoscopy process.
4. There was only a survey or questionnaire or interview used as the study method (eg, studies did not report diagnostic outcomes).

In addition, references were assessed for availability, language, and study method during the abstract selection. If the full, original paper was not available for free; the language was different from English or Dutch; or the study method was solely a survey, questionnaire, or interview, the reference was excluded. References were also excluded if they were not an original study (eg, conference abstract or letter [to the editor]). The included references were mapped with those of references from the included systematic reviews for validation of the search query.

### Descriptive Content Analysis of Heterogeneous Factors

A descriptive content analysis was performed by 2 authors (FvS and APL) using the framework of study characteristics [16] (Table 1). This framework was applied to individual references included in the scoping review. Content regarding each study characteristic was extracted by one author and reviewed by the other author. Extracted content for each study characteristic was summarized and analyzed descriptively, with subsequent presentation to a third, independent author (LWP) for consensus.

**Table 1:** The framework of study characteristics contributing to heterogeneity.

Domain	Study characteristics
Population	Sample size Selected lesion Single or multiple lesions per patient Type of lesion Study setting Inclusion or exclusion criteria of patients
Index test	Profession of photographer Practitioner taking dermatoscopic images Training Experience Dermatoscopic images Quality Exclusion of images Additional clinical information
Reference standard	Profession of assessor Practitioner assessing dermatoscopic images Training Experience Gold standard

## Results

### Literature Search

**Figure 1:**
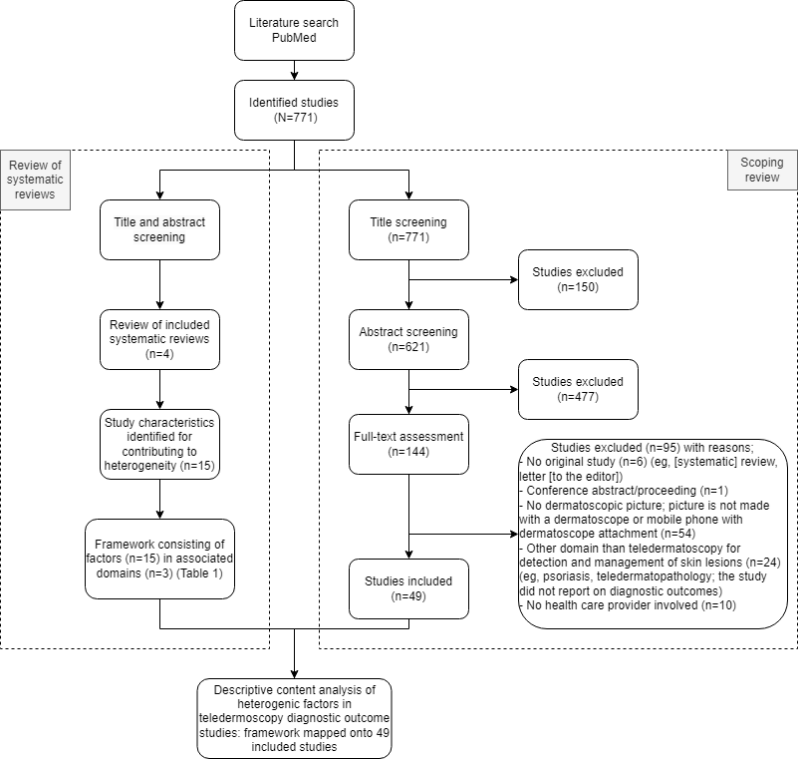
PRISMA (Preferred Reporting Items for Systematic Reviews and Meta-Analyses) flow diagram.

The applied search query can be found in Multimedia Appendix 1. This search query resulted in a total of 771 references, with no duplicates identified (Figure 1).

### Review of Systematic Reviews

Searching the cohort of references yielded 6 systematic reviews [8-12,17]. However, 2 were excluded since they did not meet the inclusion criteria. Specifically, they only focused on automated smartphone apps and the use of teledermatology during the COVID-19 pandemic [9,17]. A total of 4 systematic reviews [8,10-12] remained and were subject to an in-depth assessment, and this yielded 15 study characteristics grouped into one of the domains (population, index test, and reference standard; Table 1).

### Scoping Review

After the title and abstract scan of the 771 references, 627 references were excluded. A full-text assessment was performed for 144 references, after which 49 references were included. The reference selection process can be reviewed in Figure 1.

While checking the included references against the systematic reviews, 17 references were found once, 7 were found twice, and 3 were found 3 times. The other 33 references were not included in any of the systematic reviews, due to the more recent publication date of the references compared with the systematic reviews.

### Descriptive Content Analysis of Heterogeneous Factors

#### Population

##### Sample Size

Studies reported on their sample sizes in the number of lesions or patients. More studies had a sample size on the smaller side compared to studies with a large sample size. Of the 49 studies, 16 (33%) [18-33] had a sample size from 1-99, a total of 28 (57%) [2,6,34-59] had a sample size from 100-999, a total of 3 (6%) [60-62] had a sample size from 1000-9999, and 2 (4%) [63,64] had a sample size greater than 10,000.

##### Single, Multiple, and Types of Selected Lesions

The majority (29/49, 59%) of the studies included multiple lesions per patient, while 7 studies [25,30,38,44,51,57,61] limited this to a maximum of 1 lesion per patient. In 13 (26%) out of 49 studies [22,24,27,33,35,40,42,47,54,58,60,63,64], it was not possible to determine if the researchers considered multiple lesions per patient because they solely mentioned the total number of selected lesions without reporting on the total number of patients.

Furthermore, there was a wide variety of included lesion types across the studies. Several studies included only 1 specific (potentially complex) lesion type, while others did not select 1 specific type of lesion of interest, resulting in a variety of included lesion types per study. In addition, 8 (16%) studies [19,29,41,47,52,53,56,62] did not mention the type of lesions they included in their studies.

##### Study Setting

Data on the study setting were analyzed according to where the dermatoscopic picture was taken and the patient was examined, rather than the location where the teledermoscopy consultation was assessed. Notably, studies took place in various settings, for which studies were predominantly performed in secondary care, such as within dermatology departments at hospitals (20/49, 41%) [19,21,23,25,26,29,31-34,38,44,48,51,54,56, 57,59,62,63]. Also, studies were performed in the primary care settings (11/49, 22%) [2,20,24,37,39,40,45,47,50,58,60], such as the general practitioner facilities, and tertiary care settings (10/49, 20%) [6,18,22,28,30,36,43,46,53,55], exemplified by dermatology clinics. A total of 3 studies were performed by using only data from an electronic medical record [35,49,64], while 2 studies were part of a population screening [41,52]. Overall, 3 studies did not mention the study setting [27,42,61].

##### Inclusion and Exclusion Criteria of Patients

A total of 11 (22%) out of 49 studies did not specifically state if inclusion or exclusion criteria of patients were applied [21,25,27,30,31,33,34,55,60,61,63]. The remaining studies reported a diverse range of inclusion and exclusion criteria of patients, varying from elaborate criteria to none. The variety of these criteria made it impossible to create comprehensive categories that could summarize the criteria across different studies. The main criteria were the number or type of lesions under the study’s consideration, the age of the patient, and the study setting. For example, patients with lesions deviating from the lesion of study interest were excluded, as were patients referred from a deviating study setting than defined for the respective study.

#### Index Test

##### Profession, Training, and Experience of Photographer in Taking Dermatoscopic Images

The majority (36/49, 73%) of studies provided information on the type of professional responsible for obtaining (dermatoscopic) images. Specifically, 12 (24%) out of 49 studies reported professions directly related to dermatology, such as melanographers, specialized nurses, and dermatologists [6,18,19,23,28,32,34,36,37,42,55,56]. From this group, 3 studies reported that the practitioners were trained, of which only 1 study provided details on the duration and content of the training (eg, training course of 6 hours, clinical diagnosis of skin cancer, and using the teledermoscopic system). Notably, those 3 studies did not mention the level of experience of practitioners in taking dermoscopic images. The remaining 9 studies did not report on provided training, of which 4 (44%) also did not report on the level of experience of the trainees. Among these 9 studies, 5 (56%) reported that the practitioners were deemed experts in taking images but without further details on the level of their experience.

In 16 (33%) out of 49 studies, the reported profession of practitioners was not directly associated with the field of dermatology, for example, general practitioners, or students and doctors in training [2,20,24,29,39-41,45,47-50,52,58,60,64]. For 12 of those studies, the practitioners were trained and details were provided about the time, content of the training, or both aspects. The provided training ranged from a 1-hour long introductory training session on how to use the teledermatology system and camera to training sessions that took place over a 3-month period on best practices and how to take (dermatoscopic) images. However, few details were provided about how the training participants were trained (eg, one-on-one, classroom, and clinical simulation), and there was not much information about who the trainer was. Only 3 studies mentioned that the type of training included “learning courses, direct meetings, and involving self-assessment procedures,” “a course at the institution,” and “PowerPoint tutorial”. It was not mentioned whether the practitioners had previous experience in taking images. A total of 4 (25%) of the 16 studies did not mention the provided training and level of experience of the practitioners.

In addition, 8 (16%) of the 49 studies did not describe the profession of photographer specifically [35,43,53,54, 57,59,61,62]. For instance, “research staff” was mentioned in 2 studies, but without specifically indicating their field of specialization. Other studies mentioned “administrators,” “a member of staff who had been trained,” and “technician,” all without further specific information. None of those studies mentioned that the practitioner had previous experience in taking images, and 2 studies mentioned that the practitioner was trained, but no details were provided about training.

Finally, there were 13 (26%) of the 49 studies that did not mention the profession of the practitioner [21,22,25-27, 30,31,33,38,44,46,51,63]. Those studies also did not provide details regarding received training by the practitioner on taking (dermoscopic) images. Only 2 studies mentioned that the practitioner was an expert in taking (dermoscopic) images without providing details on the level of experience.

##### Image Quality and Exclusion of Images

A total of 23 (47%) of the 49 studies did report on an image quality assessment in various manners [2,19-24,28-30,32,34,36, 44,45,47,48,50,52,56,62-64]. Four (17%) of those 23 studies did not specifically mention the number of images with the corresponding quality assessment such as “good” or “poor”. They reported that (some) poor-quality images were observed, or that most images were of excellent or good quality. The remaining 19 (19%) studies reported both the quality assessment (eg, good or excellent) along with the corresponding number of images. Of these 19 studies, 5 (26%) reported the image quality on a 3-point scale (eg, low, moderate, or excellent quality). Overall, 6 (26%) out of 23 studies reported the number of images that were excluded due to poor quality, and 1 study only reported that poor-quality images were excluded without specifying the number of images. One study mentioned that image quality was not assessed in the study [31].

In total, 25 (51%) out of 49 studies did not report on the image quality, and 3 of these studies mentioned that images were excluded [6,18,25-27,33,35,37-43,46,49,51,53-55,57-61]. The number of excluded images varied from 4 to 149 per study for either a poor-quality assessment or “various reasons”.

##### Additional Clinical Information

Besides providing the (dermatoscopic) images in the teledermoscopy consultation, 35 (71%) out of 49 studies also provided additional clinical information [2,6,11,18,19,21,25,26, 29-37,40-46,48-50,53,55,56,58-62]. This could include, for example, the patient’s demographic information, lesion characteristics, medical history, histopathology results, treatment, and diagnosis. The majority of studies provided a combination of these topics. Demographics, medical history, and lesion characteristics were mostly provided, followed by risk factors and histopathology results.

There were 12 (24%) out of 49 studies that did not mention if any additional clinical information was added to the teledermoscopy consultation [20,23,24,27,28,38,39,47,52, 54,63,64]. A total of 2 (4%) out of 49 studies mentioned that no additional clinical information was added to the teledermoscopy consultation [22,51].

#### Reference Standard

##### Profession, Training, and Experience of Practitioner in Assessing Dermatoscopic Images

The majority (44/49, 90%) of studies reported that the teledermoscopy consultation was assessed by a dermatologist or teledermatologist [2,18-21,23-42,45-62,64]. For one of these studies, it was mentioned that the practitioners (dermatologists and plastic surgeons) received training consisting of e-learning courses, meetings, and self-assessment procedures. One other study mentioned that dermatologists were untrained, and all other studies did not mention anything about training in assessing teledermoscopy consultations. A total of 2 studies mentioned that the dermatologists had interest and experience in skin cancer and dermoscopy; however, the teledermatologists did not have previous experience in performing teledermoscopy. The remaining studies reported that the dermatologists were experienced, but the level of experience in assessing dermatoscopic images was not reported by all the studies. The experience was either reported on a 3-point scale (eg, low, medium, or high), or the number of years was provided or other details were provided, such as “board certification”. One study reported the number of publications in dermoscopy. A total of 16 studies did not mention anything about the experience of the dermatologists.

In the remaining 5 (10%) out of 49 studies [6,22,43,44,63], the teledermoscopy consultations were assessed by a variety of professionals other than a dermatologist. For example, in 3 of the studies, the teledermoscopy consultations were assessed by plastic surgeons. They had 8-15 years of experience within their medical specialty and for one study it was mentioned that the plastic surgeons had a specific competence in the diagnosis and treatment of melanoma. In 2 (40%) of those 5 studies, the plastic surgeons received a dermoscopy course and e-learning. No further details were reported regarding the duration of these training programs. Also, professions, such as an oncologist, internal medicine specialist, investigators, observer from the department of medical oncology, and independent teleconsultants, were reported in some of the studies. For all of these studies, no training details were reported. For one study, no details about the experience of the practitioner were reported, and the remaining studies reported experience on a 3-point scale (eg, beginner, average, or excellent).

##### Gold Standard

The majority (26/49, 53%) of studies applied histopathology as a gold standard to validate the diagnostic outcomes of teledermoscopy [6,18-21,25,27,31,32,36,37,39,41,43,44,46,49, 51,54,57-61,63,64]. A total of 14 (29%) out of 49 studies [2,28-30,33-35,42,45,47,48,50,52,56] used both histopathology and a face-to-face consultation as a gold standard, while 6 (12%) studies only used face-to-face consultation [22,23,26,38,40,62]. One study [55] reported that diagnostic agreement between dermatologists was used as a gold standard and 2 studies [24,53] did not report what the gold standard was.

## Discussion

### Principal Findings and Implications

This scoping review assessed the variability and consistency of heterogeneous study characteristics in teledermoscopy diagnostic outcome studies. These study characteristics were retrieved from systematic reviews and grouped into the population, index test, and reference standard domains. A substantial portion of the studies did not report on study characteristics or provide limited and highly variable information. Due to the heterogeneity of the study designs, the analyzed systematic reviews could not draw definite conclusions about the accuracy of diagnostic outcomes, while these are of utmost importance to safeguard the reliability of teledermoscopy implementation [65,66]. The main findings and recommendations for future research have been summarized in Textbox 1.

**Main findings and implications for future research.**

**Main Findings**

- Population
  Sample sizes: Almost a third of the studies included fewer than 99 patients or lesions. Small sample sizes are likely to cause imprecise outcomes with wide confidence intervals.
  Lesion types: Diversity of inclusion of lesion types; high risk and common skin conditions. This diversity can impact diagnostic outcomes.
- Index test
  Image quality: Image quality can influence diagnostic outcomes. Different scales for image quality evaluation used which hinders image quality comparison across studies. Poor-quality images often excluded. Some studies provided clinician training in taking images.
- Reference standard
  Practitioner expertise: Variation in professions assessing images (dermatologists, primary care physicians, dermatoscopic readers), with unclear extent of dermoscopic knowledge or certification, and diversity in diagnostic agreement.
  Practitioner experience: Variance in years of teledermoscopy experience.

**Implications for Future Research**

- Challenges
  Heterogeneity and limited reporting hinder assessment of study evidence.
  Systematic reviews remain cautious due to study variability.
- Checklists
  Suggested use of tailored STARD (Standards for Reporting Diagnostic Accuracy Studies) checklists to improve reporting quality.
- Future
  Development and implementation of tailored checklists will be time-consuming.
  Complete reporting essential before applying tools like QUADAS-2 (Quality Assessment of Diagnostic Accuracy Studies-2) for bias assessment and a systematic review.

### Main Findings in Relation to the Literature

#### Population

Almost one-third of the studies included fewer than 99 patients or lesions. Such small sample sizes could lead to imprecise diagnostic outcomes with wide confidence intervals [67]. Larger sample sizes generally lead to increased statistical power and more precise study outcomes. This highlights the need for future research with larger sample sizes to enhance the reliability of diagnostic outcomes in teledermoscopy. Perhaps, small sample sizes were due to medical-ethical concerns or low incidences of lesions being studied. However, these studies did not report on their considerations for a relatively small study sample size.

Furthermore, some included lesion types represented a high-risk population, such as lesions suspected of being melanoma, while others reflected common skin conditions found in the general population. This diversity can significantly impact diagnostic outcomes, since complex lesions pose greater diagnostic challenges compared with common lesion types. Hence, understanding the types of lesions included in a study is crucial for interpreting the diagnostic outcomes. Studies by Piccolo et al [30] and Wang et al [68] highlighted that diagnostic difficulty, rather than image quality, correlates with diagnostic accuracy, particularly in the case of pigmented skin lesions. So, image quality may not be the sole factor influencing diagnostic outcomes, especially when dealing with complex lesions.

#### Index Test

Image quality is an important study characteristic in teledermoscopy studies, as it could have influenced the diagnostic outcomes. While most included studies evaluated image quality on a 3-point scale (eg, low, medium, or excellent), others simply counted “good” images for diagnosis. However, there was inconsistency in the scales used in each study, and clear definitions for the terms “poor,” “out of focus,” or “excellent” were not described. This hinders image quality comparisons across studies. Some studies excluded low-quality images, while in one study, the researcher decided to physically refer those cases to a dermatologist [69]. In general practice, poor photo quality will likely lead to unnecessary patient referrals.

Studies identified efforts to improve the image quality, such as clinician training in taking (dermoscopic) images [32,45,52,62,70,71]. However, even when such training was provided, there were still instances where poor-quality images were obtained. This was often due to the differing educational backgrounds of professionals (eg, physician or nurse), equipment, and training. Up to 12% of the images in these studies were still of poor quality. van der Heijden et al [45] reported that 36% of the images were of poor quality, despite general practitioners taking part in a 1-hour training session on the teledermoscopy system, camera, and dermatoscope. Indeed, the diagnostic accuracy improved with high-quality images.

In practice, the National Health Service (NHS) has developed standards for teledermatology practice and recommendations for photographers and presented them in their teledermatology roadmap [72]. The NHS places specific attention on image quality (setting standards for taking high-quality images), what clinical information is required, and outlining qualifications for photographers and clinicians who will review the images. This is a good example of providing support to teledermatology users to implement effective teledermatology and accelerate the roll-out of teledermatology.

#### Reference Standard

Remarkable differences emerged in study outcomes based on the profession of the practitioner who took or assessed the (dermoscopic) images in teledermoscopy consultations. Besides dermatologists, primary care physicians, and dermatoscopic “readers” were involved. However, the extent of their dermoscopic knowledge or certification was not clear from our review.

These uncertainties surrounding the assessors’ type of expertise suggest there is a need to assess who should be undertaking teledermoscopy consultations to obtain accurate diagnostic outcomes. This raises the question as to whether other professionals besides dermatologists are adequately experienced in the use of teledermoscopy tools in their practice and how expertise influences teledermoscopy consultations. This is also reflected in the teledermatology systematic review of Bourkas et al [73], where results showed that nonspecialists showed significantly lower agreement among nonspecialists compared with teledermatologists. In addition, the variety of professions involved might not represent those using teledermoscopy tools in general practice. In some countries, dermatologists are expected to diagnose cancerous lesions through teledermoscopy, while in other countries, general practitioners may do so. Therefore, this study suggests that future studies also include the different modalities for doing teledermoscopy, ranging from a general practitioner taking pictures to a dermatologist, and so on.

Furthermore, there was variance in the dermatologists’ years of teledermoscopy experience. Notably, Kittler et al [74] reported that dermoscopy improves the diagnostic accuracy for melanoma compared with an unaided eye examination, but this effect was observed among experienced dermatologists only. The findings described above emphasize the need to recognize that teledermoscopy is highly dependent on good-quality images, and consistent reporting is needed in studies. Moreover, many teledermoscopy studies have been undertaken without considering the dermatologists’ training, experience, and expertise, which are also factors closely related to diagnostic outcomes. The reference standard (level of expertise and years of dermoscopy or teledermoscopy experience) is important in safeguarding patients by ensuring that malignant lesions are not missed and having trust in the teledermoscopy system. Therefore, reporting on these factors is essential so that this can be taken into account in systematic reviews to assess the evidence.

Furthermore, an analysis of the conclusions of the included studies reveals predominantly positive attitudes toward the use of teledermoscopy in practice. This contradicts with the more critical opinion of the authors of the systematic reviews, who refrain from a definite conclusion due to the study heterogeneity. Recommendations for improvements made by studies included enhancing the image quality, larger sample sizes, and guidelines for the use of teledermoscopy in practice. Thus, this addresses again the need for homogeneous studies to allow meaningful comparisons by systematic reviews.

#### Implications for Future Research

In this study, we have addressed the challenges posed by heterogeneity and the limited details available about study characteristics in teledermoscopy diagnostic outcomes studies. Without full and transparent reporting, researchers are unable to assess the evidence of individual studies as well as to perform systematic reviews and meta-analysis. This hinders the translation into practice guidelines [13]. Currently, systematic reviews are still cautious in their conclusions.

#### Use of Checklists

The use of checklists, such as the STARD (Standards for Reporting Diagnostic Accuracy Studies) [75], is suggested to serve as a tool for researchers to improve completeness and transparency in reporting of teledermoscopy diagnostic outcome studies. While the STARD checklist may not address the issue of heterogeneity directly, it provides a framework for improving and assessing the reporting quality.

The STARD checklist is a generic tool to improve the reporting in studies and thus not specifically tailored for teledermoscopy diagnostic outcome studies. Therefore, additions to suit the nuances of teledermoscopy are suggested, such as inclusion and exclusion criteria of the participants (eg, details on skin type, type, and the number of lesions) and reference standard domain (eg, level of expertise and training of photographers). It is expected that a tailored STARD checklist will enhance transparency and facilitate consistent reporting across studies.

To develop a tailored STARD checklist, a Delphi study could be conducted to gather consensus on the tailored content. We emphasize that our study characteristics will be taken into account in these discussions as a first starting point. A Delphi study will facilitate a structured and iterative process to obtain the consensus, and we suggest involving a panel of STARD/methodology experts, teledermoscopy researchers, and dermatologists to combine clinical and methodological expertise. We would suggest involving practitioners with various levels of expertise in their domain. A similar method has been applied to adapt the QUADAS-2 (Quality Assessment of Diagnostic Accuracy Studies-2) tool, resulting in a QUADAS-C (Quality Assessment of Diagnostic Accuracy Studies Comparative) tool for the assessment of comparative diagnostic accuracy studies [76]. Another example includes the STARDdem checklist, which is an elaborated STARD checklist to guide the reporting of studies of cognitive disorders. It is expected that the STARDdem checklist will increase the transparency and contribute to greater adherence to methodologic standards in studies of cognitive disorders [77]. Unfortunately, the development of these guidelines, along with the implementation of homogenous studies and subsequent systematic reviews, is a time-consuming process before it will reach general practice.

This is the first step in addressing the knowledge gap of study heterogeneity among teledermoscopy diagnostic outcome studies. For this reason, we have performed a scoping review rather than a systematic review with a QUADAS-2 analysis. Instead, we suggested the STARD checklist, a tool for checking the complete reporting of studies, which aligns with the aim of our scoping review, which is to prioritize the assessment of heterogeneous study characteristics. Before the included studies can be checked for bias using the QUADAS-2 tool, the reporting of original studies must be complete. Conducting a QUADAS-2 analysis at this stage would likely result in many “unclear” scores, making it difficult to determine potential bias. Hence, our focus was on understanding and categorizing study characteristics that have been reported for contributing to heterogeneity in teledermoscopy diagnostic outcome studies. This will facilitate future systematic reviews and bias assessments of these types of studies.

#### Strengths and Limitations

For this scoping review, we followed the published PRISMA-ScR guide; this is a published and acknowledged guideline for scoping reviews [15]. Therefore, we believe that this study was performed and reported in a comprehensive and systematic approach with a transparent and replicable review process.

An expert medical librarian was consulted to establish a search query for PubMed. This search query had a broad and inclusive scope, and, as a result, we believe that we included a wide range of relevant studies. Indeed, we could validate that a part of our included papers were included by previous systematic reviews as well. In addition, we performed an independent title, abstract, and full paper evaluation by 2 authors. However, a limitation of this study is the sole inclusion of papers published in English or Dutch and available on PubMed. This may have excluded other important studies not meeting these criteria.

Although we believe that the descriptive content analysis of the heterogeneous factors of the included studies was consistent and, objectively, this method has limitations and potential biases. It is possible that we may have misinterpreted the content of the included studies or even missed content to include. However, we believe that we have minimized these limitations and biases as much as possible by conducting the content analysis independently by 2 authors and having it reviewed by a third author.

### Conclusion

The scoping review highlights clinical and methodological heterogeneity among teledermoscopy diagnostic outcome studies, revealing considerable variability and inconsistencies in reported study characteristics. Notably, this heterogeneity is prominent in the population, index test, and reference standard domains, indicating a lack of standardized reporting. This deficiency in reporting and heterogeneity in study characteristics pose a challenge in objectively interpreting the true diagnostic outcomes of teledermoscopy. The high variability and inconsistency in reporting hinder the synopsis of diagnostic outcomes of teledermoscopy in systematic reviews, and this in turn ultimately diminishes the ability to translate teledermoscopy into routine use in general practice.

To address these challenges, it is recommended that studies adhere to the (tailored) STARD reporting guidelines. In addition, we suggest performing a Delphi study to gather consensus on the tailored STARD guideline. By promoting standardized reporting practices, this will enhance the reproducibility of study findings and improve the reliability of systematic reviews by facilitating meaningful comparisons of study outcomes. This will ultimately enhance the confidence in teledermoscopy diagnostic outcomes and support its effective integration into clinical practice.

## Appendix

Multimedia Appendix 1 Search query in PubMed.

Multimedia Appendix 2 PRISMA-ScR (Preferred Reporting Items for Systematic Reviews and Meta-Analyses extension for Scoping Reviews) checklist.

## Abbreviations

NHS: National Health Service
PRISMA-ScR: Preferred Reporting Items for Systematic Reviews and Meta-Analyses extension for Scoping Reviews
STARD: Standards for Reporting Diagnostic Accuracy Studies
QUADAS-2: Quality Assessment of Diagnostic Accuracy Studies-2
QUADAS-C: Quality Assessment of Diagnostic Accuracy Studies Comparative
