# Supplementary material for: Challenges in Teledermoscopy Diagnostic Outcome Studies: Scoping Review of Heterogeneous Study Characteristics
Source: JMIR Dermatol. 2024 Oct 18;7:e60346. doi: 10.2196/60346 (PMC11530724; doi:10.2196/60346)
Supplement: Multimedia Appendix 1 [file derma_v7i1e60346_app1.docx]

(telederm*[tiab]

OR

((*teleconsult* [tiab] OR econsult* [tiab] OR telediagnos* [tiab] OR tele-diagnos* [tiab]*

*OR consultation [tiab] OR tele-consultation [tiab]*)

AND

(dermascop*[tiab] OR dermoscop*[tiab] OR dermotoscop*[tiab] OR dermatoscop*[tiab]

OR "Dermoscopy"[Mesh] OR Surface Microscop*[tiab] OR Epiluminescence Microscop*[tiab] OR incident light microscopy[tiab] OR oil immersion diascopy[tiab]))

OR

(("Telemedicine"[Mesh] OR telehealth[tiab] OR ehealth[tiab] OR telemedicine[tiab])

AND

(dermascop*[tiab] OR dermoscop*[tiab] OR dermotoscop*[tiab] OR dermatoscop*[tiab] OR "Dermoscopy"[Mesh] OR Surface Microscop*[tiab] OR Epiluminescence Microscop*[tiab] OR incident light microscopy[tiab] OR oil immersion diascopy[tiab]))

)

AND

("Neoplasms"[Mesh] OR Melanoma* [tiab] OR Melanocyt* [tiab] OR Lesion*[tiab] OR Pigment*[tiab] OR Tumour* [tiab] OR Tumor* [tiab])
